# Health-Related Quality of Life as Measured with EQ-5D among Populations with and without Specific Chronic Conditions: A Population-Based Survey in Shaanxi Province, China

**DOI:** 10.1371/journal.pone.0065958

**Published:** 2013-07-02

**Authors:** Zhijun Tan, Ying Liang, Siming Liu, Wenjun Cao, Haibo Tu, Lingxia Guo, Yongyong Xu

**Affiliations:** 1 Department of Health Statistics, and the Ministry of Education Key Lab of Hazard Assessment and Control in Special Operational Environment, Fourth Military Medical University, Xi'an, Shaanxi Province, China; 2 Department of Economics and Finance, School of Social Sciences, Brunel University, London, United Kingdom; 3 Institute for Cardiovascular Disease, Chang Zhi Medical College, Shanxi, China; 4 Division of Medical Service, Xijing Hospital, Fourth Military Medical University, Xi'an, Shaanxi Province, China; 5 Center of Health Statistics, Health General Office of Shaanxi Province, Shaanxi Province, China; Johns Hopkins Bloomberg School of Public Health, United States of America

## Abstract

**Introduction:**

The aim of this study was to examine health-related quality of life (HRQoL) as measured by EQ-5D and to investigate the influence of chronic conditions and other risk factors on HRQoL based on a distributed sample located in Shaanxi Province, China.

**Methods:**

A multi-stage stratified cluster sampling method was performed to select subjects. EQ-5D was employed to measure the HRQoL. The likelihood that individuals with selected chronic diseases would report any problem in the EQ-5D dimensions was calculated and tested relative to that of each of the two reference groups. Multivariable linear regression models were used to investigate factors associated with EQ VAS.

**Results:**

The most frequently reported problems involved pain/discomfort (8.8%) and anxiety/depression (7.6%). Nearly half of the respondents who reported problems in any of the five dimensions were chronic patients. Higher EQ VAS scores were associated with the male gender, higher level of education, employment, younger age, an urban area of residence, access to free medical service and higher levels of physical activity. Except for anemia, all the selected chronic diseases were indicative of a negative EQ VAS score. The three leading risk factors were cerebrovascular disease, cancer and mental disease. Increases in age, number of chronic conditions and frequency of physical activity were found to have a gradient effect.

**Conclusion:**

The results of the present work add to the volume of knowledge regarding population health status in this area, apart from the known health status using mortality and morbidity data. Medical, policy, social and individual attention should be given to the management of chronic diseases and improvement of HRQoL. Longitudinal studies must be performed to monitor changes in HRQoL and to permit evaluation of the outcomes of chronic disease intervention programs.

## Introduction

Chronic diseases are the leading health challenges of the 21^st^ century. The World Health Organization (WHO) reported that, out of 36 million deaths that occurred in 2008, almost two thirds were due to chronic conditions, such as cardio-cerebral-vascular disease, hypertension, chronic respiratory disease, and diabetes. Nearly 80% of these deaths occurred in middle- and low-income countries, populations and communities [1]–[3]. Chronic diseases have become a major public health problem in China, where nearly 260 million chronic disease cases have recently been diagnosed. Along with the accelerating process of industrialization, urbanization and aging, China's health system will face multiple challenges from the increasing prevalence of chronic disease and mortality rates and from the increasing incidence of these conditions among younger and lower-income individuals [4]–[9]. Shaanxi is a middle-income province located in Northwestern China. The 2008 National Health Service Survey (NHSS) of Shaanxi Province reported that more than 6.7 million individuals, about 17.5% of the total population, had been diagnosed with chronic conditions. The incidences of many chronic diseases have increased rapidly since 2003. For example, the number of people with hypertension, diabetes, and cardio-cerebral-vascular diseases has been found to be 1.3, 1 and .30 times greater than that in 2003, respectively [10].

Chronic diseases not only pose a heavy economic burden on the individual, family, and society, but also seriously affect patients' quality of life. Studies performed during the past few decades have indicated that health-related quality of life (HRQoL), which refers to physical, psychological, and social functioning as reported by the patients themselves, has become an important component of chronic disease evaluation and monitoring in addition to conventional objective indicators, such as morbidity, mortality and clinical measurements [11]–[14]. The questionnaires, including the EuroQol questionnaire (EQ-5D), SF-36, SF-12, the Health Utilities Index (HUI) and other measurement systems, have been used in large surveys to measure HRQoL in both general and specific populations [15]–[18].

Because of the ease with which it can be administered, scored, and interpreted, EQ-5D has gained widespread popularity, especially in large-scale face-to-face health surveys conducted by interview. It also imposes a minimal burden on the respondents because it is brief and easy for most people to understand and to complete [15], [19]–[21]. The EQ-5D consists of five dimensions, mobility, self-care, usual activities, pain/discomfort, and anxiety/depression. Each dimension has three levels of response or severity (no problems, some problems and extreme problems). The ability to convert the self-classifications into a single index score makes the EQ-5D practical for both clinical and economic evaluations [15]. Unfortunately, there is no locally appropriate standard for calculation of the index. In addition to those five dimensions, the visual analog scale (VAS) component of the last part of the EQ-5D allows respondents to place their current health status on a range from 0 (worst imaginable health status) to 100 (best imaginable health status). EQ-5D population health studies have been performed in Europe, the U.S., Canada and Asia (including China) [22], [23]. Previous studies performed in mainland China and on other Chinese populations abroad have demonstrated the applicability of EQ-5D in measuring health status in Chinese populations [23]–[26]. Most previous studies have suggested that the EQ-5D is not only useful for evaluating general populations, but also applicable to various chronic diseases such as cardiovascular diseases [27], chronic heart failure [28], chronic kidney disease [29], chronic obstructive pulmonary disease [30], musculoskeletal diseases [31] and type 2 diabetes [32], [33]. In 2008, the EQ-5D was included in the NHSS for the first time in order to assess population health status in 31 provinces in mainland China. Shaanxi Province was included as a western extended sample in the 2008 NHSS.

Most EQ-5D studies performed on Chinese populations with chronic conditions have focused on one specific disease. Few have compared the impacts of different chronic diseases at the population level. In this study, the population of Shaanxi was profiled to establish a baseline for future evaluation and surveillance. The EQ-5D results were then evaluated among general patients, healthy individuals, individuals with chronic diseases and individuals with co-morbidities and the non-chronic groups were compared to those with 13 specific chronic diseases. The effects on VAS from each chronic disease and the number of chronic conditions, excluding multiple confounding effects, were evaluated.

## Methods

### National Health Service Survey

Data were collected from the Shaanxi's fourth NHSS. The NHSS is organized by the Ministry of Health (MOH) of China and it has been conducted every five years since 1993. An introduction to perform face-to-face interviews for the National Health Service Survey was provided by the MOH. The interviewers were recruited from populations of local health workers and trained by supervisors who were recruited from local health authority staff and trained at the national level. In addition, nine western provinces also use the multi-stage stratified cluster sampling method to select provincially representative samples. We adopted the NHSS form with the same questionnaire, survey and quality control methods and conducted the survey at the same time of the same year as the national level [34]. The questionnaire used for the NHSS in 2008 included more than 200 questions. These were related to the areas of acute diseases and injuries, chronic and other diseases, hospitalizations, health-related behaviors, educational level, family income and employment status, social relations, safety and security, medical care fees, accessibility of medical care (distance and time), level of satisfaction with health services, insurance coverage, vaccination and disease control, and health services specific to women and children. The EQ-5D was placed at the beginning of the questionnaire, after the sections with questions on general information and diseases and injuries, but before questions regarding health-related behavior. This was the first time that the NHSS included EQ-5D, which was useful in establishing an HRQoL baseline for the Chinese population at the national level.

In Shaanxi's NHSS 2008, the estimated sample size was 13600 according to the NHSS sample rate (1/5000), population size of Shaanxi Province (40 million) and design effect (1.7). In the first stage, to ensure the sample representativeness in the terms of demographic, socioeconomic and health status indicators, 40 out of 107 primary sample units (districts in urban areas and counties in rural areas) were randomly selected. In the second stage, 2 sub-districts (known as jiedao in Chinese) within districts and townships (known as xiang and zhen, respectively, in Chinese) were randomly selected from each of the first-stage samples. In the third stage, 2 residential committees (known as juweihui in Chinese) in the sub-districts or villages (known as cun in Chinese) of townships were randomly selected from each of the second-stage samples. If the population of the jiedao was very large, sometimes 3 or 4 committees would be selected. In each residential committee and village, 35 households were randomly selected and all family members in each sampled household were interviewed individually. EQ-5D was then reported. If participants had the ability to answer the questions in writing, they personally filled out the forms, following the instructions; otherwise a trained interviewer would collect the necessary data from respondents through face-to-face interviews.

### Classification of chronic conditions

The chronic conditions were self-reported and the interviewers had to make sure the chronic conditions were clinically diagnosed in order to reduce mistake due to self-reporting. The diagnoses and morbidity data were coded according to ICD-10 and the disease classification of the fourth NHSS [34]. Thirteen chronic diseases were extracted from the codes on the basis of both prevalence and disease burden: infectious diseases, cancer, diabetes, anemia, mental disease, heart disease, hypertension, cerebrovascular disease, respiratory disease (mainly chronic pharyngitis, pulmonary emphysema, chronic obstructive pulmonary disease, and asthma), digestive diseases (mainly anxious chronic gastroenteritis, peptic ulcers, gallstones, and cholecystitis), genitourinary disease (mainly nephritis, prostatic hyperplasia, and breast diseases), musculoskeletal disease (mainly rheumatoid arthritis, lumbar intervertebral disk diseases, and other musculoskeletal diseases), and other chronic diseases. Conditions were numbered based on the first reported chronic condition, which was considered the most important one as defined by the NHSS questionnaire.

### Classification of population

Based on the answers to the following two questions, “Have you suffered from any chronic diseases during the last half a year?” and “Have you experienced any discomfort during the two weeks immediately before the survey?”, we divided the respondents into four categories: people with chronic conditions who had experienced discomfort during the two weeks immediately before the survey, people with chronic conditions who did not experience any discomfort during the two weeks immediately before the survey, people without chronic conditions who experienced discomfort during the two weeks immediately before the survey, and people without chronic conditions who did not experience any discomfort during the two weeks immediately before the survey. The first two categories were then defined as our research population group (chronic disease group), and the third category was classified as the general patients group, and the fourth category as the healthy group. The third and fourth groups were used as reference groups.

### Statistical analysis

For convenience, certain parts of the socio-demographic variables were reclassified. For example, age was divided into three groups, (specifically 15–44, 45–64, and 65+), educational level was divided into two groups (below high school level and high school level and above), and the frequency of weekly exercise was divided into three levels (never, 1–6 times per week, more than 6 times per week). EQ-5D has ceiling effects in measuring the general population, thus we transformed the three categories of original responses in the EQ-5D self-classifier (no problem, some/moderate problems, and extreme problems/unable to) into two categories (reporting no problem and reporting any problem) [17].

Socio-demographic characteristics were examined. EQ-5D results were also obtained for different groups, such as the four groups defined in the classification of population section. The relative risk of individuals with specific chronic diseases reporting any problem against the two reference groups was analyzed based on the Chi-square test and odds ratios (ORs) and 95% confidence intervals (95% CIs) were obtained.

The independent effects of different chronic diseases, the number of chronic conditions per participant, socio-demographic variables, and health-related behavior variables were analyzed by constructing two multivariable linear regression models. The EQ VAS score served as the dependent variable for both models. In the first model, different chronic diseases, socio-demographic variables, and health-related behavior variables served as independent variables. In the second model, the number of chronic conditions, socio-demographic variables, and health-related behavior served as independent variables. All statistical analyses were implemented using SAS 9.1 for Windows, considering the complex sampling features with setting city as stratum and county as cluster.

### Ethical statements

The NHSS 2008 was approved by the National Bureau of Statistics (license number: 2008 (18)). The NHSS is implemented by the MOH every five years and the MOH has pledged to protect the privacy of respondents and to facilitate the anonymous analysis of the data according to article 14 of the third chapter of the Statistics Law of the People's Republic of China. For these reasons, written consent was not required. The investigators read the informed consent statement written on the second page of the questionnaire and verbally asked for the interviewees' permission. Once the interviewees or their caregivers or guardians (in the case of children) had verbally consented to participate in this study, the investigators would sign their own names at the beginning of the questionnaire.

## Results

Out of 13,014 respondents examined in the Shaanxi's NHSS in 2008, 1,840 were younger than 15 years old, 134 did not report in any of the five dimensions, and 53 did not answer the question “Have you experienced any discomfort during the two weeks immediately before the survey?” These respondents were excluded. Among those remaining, the rate of missing information was lower than 2%. A total of 10,987 respondents were included in the univariate analysis and 10,267 were included in the multivariable linear models.

The socio-demographic and health-related characteristics of research subgroups are shown in **Table 1**. The prevalence of chronic diseases among respondents over the age of 15 was 16.73%, 19% of whom lived with two or more chronic conditions simultaneously. The five most prevalent chronic diseases in Shaanxi Province were hypertension, musculoskeletal disease, digestive disease, heart disease, and respiratory disease. Diabetes is the cause of many other chronic diseases, the prevalence of which was also high in this study. The chronic disease groups were more likely to include older people, women, people with less education, people with lower employment rates, and people who were less active. Chronic diseases, other than hypertension, heart disease, and diabetes, were also associated with rural residents.

**Table 1:** Demographic characteristics and health-related behaviors of study subgroups.

Sample (n)	Mean age (SD)	Female (%)	Urban area	Currently married (%)	High school education or more (%)	Employed or self-employed (%)	Statutory insurance (%)	Non-smoker (%)	Non-drinker (%)	Sedentary (%)
Overall (10987)	44.3 (17)	50.4	50.0	73.7	32.1	54.4	85.3	72.0	91.0	66.7
Reference group I* (8492)	46.6 (15.8)	45.9	49.9	76.2	24.2	61.2	86.4	69.7	87.2	69.2
Reference group II* (657)	41.3 (16.3)	50.4	49.0	72.3	34.8	55.6	84.7	72.2	90.8	66.8
Infectious disease (21)	45.4 (13.9)	42.9	19.1	71.4	38.1	57.1	95.2	76.2	100.0	81.0
Cancer (17)	56 (13.3)	50.0	75.0	81.3	18.8	43.8	87.5	87.5	100.0	81.3
^2^Diabetes (97)	62.6 (11.1)	80.9	47.9	79.8	24.5	23.4	80.9	71.3	94.7	27.7
Anemia (36)	51.6 (15.7)	24.1	58.6	79.3	10.3	65.5	93.1	75.9	93.1	89.7
Mental disease (26)	48.4 (15.5)	32.0	68.0	60.0	12.0	44.0	76.0	80.0	100.0	92.0
Heart disease (184)	65.2 (12.9)	67.1	63.6	74.0	24.3	24.3	85.0	80.4	97.1	56.1
Hypertension (469)	60.3 (11.6)	68.6	54.4	78.9	28.2	37.6	83.7	72.3	93.6	51.4
Cerebrovascular disease (78)	64.6 (11.0)	43.1	45.8	77.8	19.4	33.3	90.3	69.4	98.6	70.8
Respiratory disease (120)	57.9 (15.0)	36.4	40.7	79.7	22.0	62.7	89.8	60.2	89.0	78.0
Digestive disease (189)	51.7 (13.0)	39.2	51.9	84.0	21.6	68.5	93.4	62.4	91.7	76.2
Genitourinary disease (62)	54.7 (14.3)	45.2	59.7	85.5	16.1	40.3	95.2	74.2	95.2	66.1
Musculoskeletal disease (268)	52.9 (13.4)	39.0	57.1	81.1	18.5	56.8	89.2	71.4	88.8	76.5
Other chronic diseases (271)	52.6 (15.1)	44.1	57.0	80.9	18.4	55.9	87.9	76.6	92.2	73.4

* Reference group I  =  healthy population, representing the respondents without chronic disease and no apparent health problems during the two weeks immediately before the survey.

* Reference group II  =  patients without chronic conditions (general patients), representing the respondents without chronic disease but some detectable health problem during the two weeks immediately before the survey.

**Figure 1:**
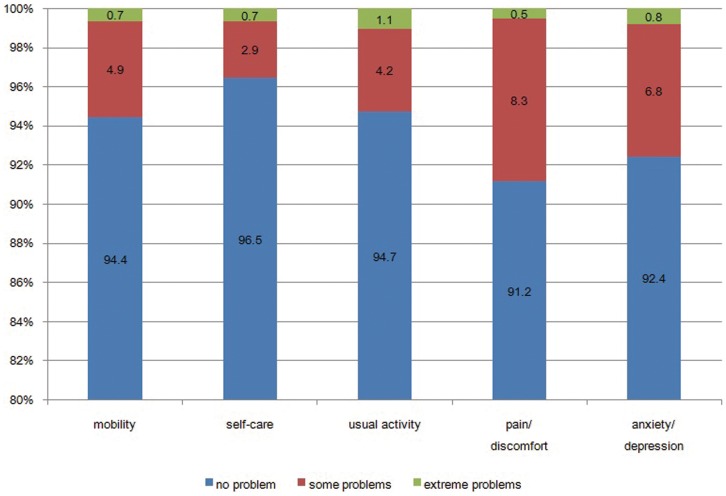
Distribution of the EQ-5D self-classified health states of the general population of Shaanxi Province, China.

As shown in Figure 1, 5.6%, 3.6%, 5.3%, 8.8%, and 7.6% of the respondents reported problems in the mobility, self-care, usual activities, pain/discomfort, and anxiety/depression dimensions of the EQ-5D, respectively. Overall, self-care dimension had the lowest association with reporting any problem, however it was most likely to be associated with reporting extreme problems (1.1%). As shown in **Table 2**, the relative number of chronic diseases among respondents who reported any problem in the dimensions of EQ-5D approached 50%, which indicated that the effects of chronic diseases on HRQoL, among residents of Shaanxi, are very serious.

**Table 2:** Proportions of population with and without chronic conditions among those who reported any problem in either dimension of EQ-5D.

	Chronic disease		No chronic disease	
EQ-5D	Number	Percentage	Number	Percentage
mobility	347	56.8	264	43.2
self-care	235	60.6	153	39.4
usual activities	339	58.5	240	41.5
pain and discomfort	550	56.6	422	43.4
anxiety and depression	406	49.0	423	51.0

### 

#### Table 3

shows the EQ-5D and EQ VAS scores of the four groups and lists the recalculated results of EQ-5D and EQ VAS scores of NHSS 2008. Results indicated that the likelihood of reporting any problem in any of the five dimensions was greatest among respondents with chronic diseases and detectable health problems during the two weeks immediately before the survey, followed by those with chronic diseases but no health problems during the two weeks immediately before the survey, then general patients, and lastly healthy people. The only exception was in the anxiety/ depression dimension, in which the general patient group was a little more likely to report problems than individuals with chronic diseases who had no reported health problems during the two weeks immediately prior to the survey (21.3% vs. 19.3%, respectively). However, this was not true for EQ VAS. **Table 4** shows the results of EQ-5D and EQ VAS among respondents with different numbers of chronic conditions. The effects on the HRQoL increased as the number of chronic conditions increased.

**Table 4:** Proportions of reporting any problem in EQ-5D and EQ VAS among patients with one or more chronic conditions.

	Total	Number of chronic condition			
		one	two	three or more	P-value
**EQ-5D**					
mobility	18.9	16.7	26.2	36.5	0.003*
self-care	12.8	11.4	18.2	20.6	0.037*
usual activities	18.4	16.5	24.8	34.9	0.007*
pain and discomfort	30	27.7	39.5	41.3	0.032*
anxiety and depression	22.1	21	25.2	34.9	0.019*
**EQ VAS**					
Mean(SE)	68.5 (1.1)	69.5 (1.2)	64.7 (2.2)	63.7 (3.2)	<0.001**

* : chi-square test whether there is any difference among any of the three chronic groups with different number of chronic condition;

** : ANOVA test; SE: standard error.

**Table 3:** Proportions of reporting any problem in EQ-5D and EQ VAS among the four groups.

	Total	Groups					NHSS 2008 [23].	
		Group 1	Group 2	Group 3	Group 4	P-value	Total	Western
**EQ-5D**								
mobility	5.6	23	14.8	10.4	2.3	<0.0001	5.2	4.8
self-care	3.6	17.2	8.6	6.9	1.3	<0.0001	3.3	3
usual activities	5.3	23.9	13.1	10.5	2	<0.0001	4.8	4.4
pain and discomfort	8.8	40.9	19.3	21.3	3.3	<0.0001	9.2	7.4
anxiety and depression	7.6	29	15.5	12.8	4	<0.0001	6.4	5.8
**EQ VAS**								
Mean(SE)	80(0.7)	65.3 (1.7)	71.6 (0.8)	76.7 (1.4)	82.7 (0.7)	<0.0001	79.8 (−)	77.6 (−)

Group 1: People with chronic conditions and had experienced discomfort during the two weeks immediately before the survey;

Group 2: People with chronic conditions but did not experience any discomfort during the two weeks immediately before the survey;

Group 3: People without chronic conditions and experienced discomfort during the two weeks immediately before the survey;

Group 4: People without chronic condition but did not experience any discomfort during the two weeks immediately before the survey.

P value: chi-square test whether there is any difference among any of the four groups.

NHSS 2008: National Health Service Survey conducted in 2008 in China. We recalculated the EQ-5D and EQ VAS of the overall population and western population according to the results reported by Sun S [23].

#### Table 5

shows the odds ratios among individuals with chronic diseases to report problems compared with each of the two reference groups. Individuals with chronic diseases were significantly more likely to report any problem in EQ-5D than healthy individuals. Cancer, mental disease, heart disease, cerebrovascular disease, musculoskeletal disease, and the other chronic diseases were found to have significant effects in all of the five dimensions when compared to participating patients lacking these conditions. Some chronic diseases showed statistically significant associations with certain dimensions, such as anemia with A/P, hypertension and genitourinary disease with MO, and respiratory disease with UA and A/P. Among the 13 chronic diseases, cerebrovascular disease, cancer, and mental disease were the three conditions with the greatest odds ratios in all five dimensions when compared to both reference groups.

**Table 5:** Risk of non-chronic problems among respondents with specific chronic diseases.

Disease	OR (95% CI)				
	mobility	self-care	usual activities	pain and discomfort	anxiety and depression
**Infectious disease**					
OR I	4.1 (1.0–16.4)	3.7 (0.4–30.9)	4.7 (1.1–19.2) *	7.1 (2.8–17.8) **	5.9 (2.0–17.0) **
OR II	0.9 (0.2–3.7)	0.6 (0.0–6.0)	0.9 (0.2–3.6)	1.1 (0.4–2.9)	1.8 (0.5–5.8)
**Cancer**					
OR I	12.7 (4.6–35.2) **	18.4 (6.5–52.0) **	14.5 (5.9–35.8) **	14.1 (7.0–28.5) **	10.2 (5.8–18.0) **
OR II	2.8 (1.0–7.7)	3.4 (1.2–9.5) *	2.8 (1.1–6.7) *	2.2 (1.1–4.3)	3.2 (2.0–5.0) **
**Diabetes**					
OR I	5.3 (2.8–9.9) **	5.6 (2.4–12.9) **	8.1 (4.7–14.2) **	7.1 (4.1–12.2) **	4.8 (2.6–9.1) **
OR II	1.1 (0.6–2.1)	1.0 (0.5–2.0)	1.5 (0.9–2.7)	1.1 (0.6–1.9)	1.5 (0.9–2.5)
**Anemia**					
OR I	4.8 (1.6–13.7) **	-	2.7 (0.7–9.8)	8.3 (4.5–15.3) **	8.3 (4.4–15.5) **
OR II	1.0 (0.3–3.2)	-	0.5 (0.1–1.8)	1.3 (0.7–2.2)	2.6 (1.3–5.2) **
**Mental disease**					
OR I	9.9 (4.9–20.3) **	27.1 (13.7–53.6) **	28.6 (16.3–50.0) **	16.1 (9.1–28.7) **	16.3 (10.8–24.6) **
OR II	2.2 (1.0–4.6) *	5.0 (2.4–10.6) **	5.4 (3.0–9.9) **	2.5 (1.4–4.5) **	5.1 (3.0–8.5) **
**Heart disease**					
OR I	10.8 (7.3–16.0) **	11.5 (6.5–20.6) **	11.1 (7.1–17.3) **	10.1 (7.0–14.6) **	7.3 (4.8–11.1) **
OR II	2.4 (1.7–3.4) **	2.1 (1.2–3.7) *	2.1 (1.3–3.3) **	1.5 (1.0–2.4) *	2.3 (1.4–3.7) **
**Hypertension**					
OR I	6.8 (5.0–9.2) **	7.7 (4.7–12.5) **	6.7 (4.7–9.6) **	6.2 (4.6–8.3) **	3.7 (2.7–5.2) **
OR II	1.5 (1.0–2.1) *	1.4 (0.9–2.0)	1.3 (0.8–1.9)	0.9 (0.6–1.3)	1.1 (0.8–1.6)
**Cerebrovascular disease**					
OR I	28.3 (20.7–38.6) **	42.2 (28.1–63.6) **	31.1 (22.3–43.5) **	19.2(14.5–25.6)**	11.8 (8.7–16.0) **
OR II	6.3 (4.6–8.5) **	7.8 (5.3–11.4) **	5.9 (4.0–8.9) **	3.0 (2.1–4.2) **	3.7 (2.7–5.0) **
**Respiratory disease**					
OR I	6.8 (4.0–11.7) **	9.8 (5.2–18.2) **	8.6 (4.9–15.0) **	8.5 (5.1–14.0) **	5.4 (2.9–10.0) **
OR II	1.5 (0.8–2.7)	1.8 (0.9–3.4)	1.6 (0.8–3.1)	1.3 (0.8–1.9)	1.6 (0.9–3.0)
**Digestive disease**					
OR I	2.7 (1.4–5.0) **	4.9 (2.4–10.0) **	4.4 (2.0–9.5) **	7.0 (4.0–12.1) **	3.1 (1.8–5.4) **
OR II	0.6 (0.2–1.3)	0.9 (0.3–2.2)	0.8 (0.3–2.1)	1.0 (0.6–1.8)	0.9 (0.6–1.6)
**Genitourinary disease**					
OR I	9.0 (5.1–16.1) **	7.6 (3.4–16.9) **	7.2 (3.4–14.9) **	7.2 (3.6–14.4) **	4.4 (2.5–7.6) **
OR II	2.0 (1.0–3.9) *	1.4 (0.6–2.9)	1.3 (0.6–3.0)	1.1 (0.5–2.2)	1.3 (0.7–2.4)
**Musculoskeletal disease**					
OR I	9.5 (6.3–14.3) **	8.7 (4.7–16.4) **	8.8 (5.4–14.5) **	11.1 (7.7–15.8) **	5.4 (3.4–8.3) **
OR II	2.1 (1.5–2.9) **	1.6 (0.9–2.6)	1.7 (1.0–2.7) *	1.7 (1.3–2.2) **	1.6 (1.1–2.5) *
**Other chronic disease**					
OR I	7.0 (5.2–9.3) **	10.4 (7.2–15.0) **	9.1 (6.6–12.6) **	9.8 (7.2–13.4) **	5.9 (4.7–7.6) **
OR II	1.5 (1.0–2.2) *	1.9 (1.2–2.9) **	1.7 (1.1–2.6) *	1.5 (1.1–2.0) **	1.8 (1.4–2.4) **

Note: OR I  =  odds ratio of respondents with the selected chronic diseases relative to reference group I as defined in Table 1; OR II  =  odds ratio of respondents with the selected chronic diseases relative to reference group II as defined in Table 1; *: chi-square test *P*<0.05; **: chi-square test *P*<0.01; No marks: not significant.

The results of multivariate linear regression models for EQ VAS are shown in **Table 6** **.** Both model I and model II accounted for about 30 percent of the total variance. The collinearity effects were small and could be ignored. Model I indicated that all of the selected chronic diseases, except anemia, were significant risk predictors for the EQ VAS score. In model II, gradient increasing effects were also observed in the chronic condition number (β = −6.52 for one condition, β = −9.89 for two conditions, and β = −10.58 for three or more conditions). These factors were not found to have significant effects in the general patient group (β = 0.66, *P* = 0.7027). As shown in Table 6, the results of both model I and model II indicated that residence in an urban area, male gender, more education, young age, and more physical activity were predictive of higher EQ VAS scores. Gradient increasing effects were found to be statistically significantly associated with age and physical activity, which meant that the HRQoL decreased with increasing age and increased with higher frequency of physical activity. Widowed individuals were more likely to report lower EQ VAS scores than those currently married and singles were likely to report higher HRQoL scores. Employment was associated with a higher EQ VAS score. Free medical service was associated with better HRQoL scores. Smoking and alcohol use were not found to be associated with significant negative effects.

**Table 6:** Results of multivariate linear regression models.

Indicator	Model I^a^			Model II^b^		
	β^c^	SE^d^	*P* value	β^c^	SE^d^	*P* value
**Chronic diseases (against people with no chronic diseases at all)**						
Infectious disease	−9.41	1.66	0.0004	-	-	-
Cancer	−13.73	5.2	0.0351	-	-	-
Diabetes	−5.86	1.28	<.0001	-	-	-
Anemia	−4.08	2.51	0.1144	-	-	-
Mental disease	−28.03	3.58	<.0001	-	-	-
Heart disease	−10.7	1.9	<.0001	-	-	-
Hypertension	−4.69	0.87	<.0001	-	-	-
Cerebrovascular disease	−17.83	1.93	<.0001	-	-	-
Respiratory disease	−4.93	2.02	0.0210	-	-	-
Digestive disease	−4.54	1.28	0.0013	-	-	-
Genitourinary disease	−7.17	1.93	0.0008	-	-	-
Musculoskeletal disease	−5.9	0.95	<.0001	-	-	-
Other chronic disease	−9.07	1.21	<.0001	-	-	-
**Co-morbidity (against healthy population)**						
No CD but health problems experienced during the two weeks before the survey	-	-	-	0.66	1.71	0.7027
One CD	-	-	-	−6.52	0.77	<.0001
Two CDs	-	-	-	−9.89	1.72	<.0001
Three or more CDs	-	-	-	−10.58	3.1	0.0018
**Socio-demographic characteristics**						
Urban area of residence	0.84	1.65	0.0254	0.90	1.68	0.0325
Male	0.84	0.35	0.0226	0.81	0.34	0.0244
High school education or more	1.08	0.39	0.0097	1.02	0.39	0.0146
Discomfort experienced during the two weeks immediately before the survey	−4.89	0.86	<.0001	−5.22	1.59	0.0027
**Age (against 65+)**						
15–44	10.82	0.93	<.0001	10.87	0.9	<.0001
45–64	5.65	0.79	<.0001	5.75	0.77	<.0001
**Marital status (against those currently married)**						
Divorced	−1.01	1.29	0.4362	−1.06	1.31	0.42
Widowed	−1.61	0.77	0.0457	−1.58	0.738	0.0394
Never married	2.49	0.61	0.0003	2.42	0.61	<.0001
Other	1.83	1.43	0.2096	1.7	1.42	0.2399
**Employment (against employed)**						
Retired	0.19	1.08	0.8601	0.31	1.1	0.7785
Unemployed	−1.83	1.55	0.0248	−1.98	1.55	<.0001
Student	0.01	0.72	0.9927	0.07	0.71	0.9276
**Social medical insurance (against those with no social medical insurance)**						
Urban workers' medical insurance	−1.77	0.99	0.0842	−1.75	1.01	0.0935
Free medical service	2.82	3.08	0.0167	2.79	2.95	0.0097
Urban residents' medical insurance	−0.17	1.14	0.8808	−0.15	1.16	0.8959
New rural cooperative medical system	−0.52	1.46	0.7217	−0.54	1.51	0.7207
Other	0.55	1.55	0.7238	0.46	1.54	0.7691
**Smoker (against lifelong nonsmokers)**						
Smoker	−0.77	0.48	0.1197	0.85	0.47	0.0790
Ex-smoker	−2.21	1.37	0.1170	−1.59	1.25	0.2150
**Alcohol use (against those who have never drunk alcohol)**						
One or two times a week	−0.92	1.22	0.4545	−1.01	0.84	0.2386
More than three times a week	0.17	1.67	0.9184	−0.08	1.69	0.9625
**Physical exercise (against those who never exercise)**						
No more than 6 times a week	1.98	0.62	0.0032	2.12	0.62	0.0019
More than 6 times a week	2.81	0.71	0.0005	2.95	0.69	0.0002
Constant	73.07	2.97	<.0001	72.90	1.76	<.0001
F Value	158.06	-	<.0001	767.49		<.0001
R-square	0.3			0.28		
Total number of respondents in the model	10267			10267		

Note: a: Model I, in which EQ VAS was the dependent variable, and 13 classes of chronic diseases and socio-demographic and health behavior variables were independent variables;

b: Model II, in which EQ VAS was the dependent variable, and co-morbidity and socio-demographic and health behavior variables were independent variables;

c: Regression coefficient (linear relationship) indicating the difference in EQ VAS score comparing each subgroup to the reference;

d: SE, standard error.

## Discussion

EQ-5D has been used in many studies to measure the HRQoL among individuals with chronic diseases. The underlying results of these previous studies have demonstrated that chronic disease is an important risk factor for low HRQoL [22]. In the current study, the associations between the chronic conditions and socio-demographic and self-perceived health that were measured using the EQ-5D were evaluated and a health-status baseline was established at the population level in Shaanxi Province. This was done to create a normative database for use as a reference when conducting further studies regarding HRQoL, in populations with chronic disease, in this region.

The results indicated that nearly half of the respondents who reported any problem in the dimensions of EQ-5D had lived with clinically diagnosed chronic conditions at some point in their lives. This suggests that chronic diseases are becoming some of the leading risk factors for health status in Shaanxi Province. The EQ-5D and EQ VAS score results were similar to those of the national and western populations in the NHSS 2008. Pain/discomfort and anxiety/depression were the two most frequently reported dimensions with some or extreme problems, which was what was found in the NHSS 2008 as well. All participants with chronic conditions were significantly more likely to report any problem in the dimensions of EQ-5D than healthy individuals. Individuals with chronic diseases, including mental disease, cerebrovascular disease, cancer, and heart disease, were significantly more likely to report any problem than patients with non-chronic conditions. This held true across all five dimensions. These chronic diseases involve serious impairments at the individual level, even though they are not the most prevalent chronic diseases [26], [35]. For example, mental disease is less prevalent than physical diseases are, but it was found to be significantly more closely associated with reports of other problems in all of the dimensions when compared with the two reference groups. The negative effects of mental disease on EQ VAS were the largest (coefficient = −28.03, *P*<0.0001). Therefore, impairments that each chronic condition has on health at population level, as well as the individual level, should be considered. Mental disease is often difficult to detect and diagnose due to the delicacy of the issue in Chinese culture [36], [37]. For this reason, the prevalence and the level of impairment brought on by mental disease may have been underestimated in this study.

Results showed that, with the exception of anemia, all of the chronic diseases evaluated in this study were risk factors for low EQ VAS scores. Statistically significant factors involving socio-demographic and health behavior variables included age, education level, marital status, location of residence, employment status, and frequency of physical activity. In a previous studied, Pappa suggested that medical insurance had a significant protective effect on health [14]. However, in our study, we did not find that the Urban Residents' Medical Insurance and/or the New Rural Cooperative Medical System, which are the main providers of social health insurance in China, had any positive effects on the EQ VAS scores. This indicates that the health service provided by the current main social medical insurance providers should be further extended and improved. The protective effect of free medical service was statistically significant. However, this insurance plan covers only certain people in China, most of whom are government officers, and these people consume a great deal of health resources freely. This is indicative of inequality in health insurance policy [38], [39]. Smoking and alcohol use had no significant negative effects on the EQ VAS score, and this may have been due to measurement bias and sampling errors.

In this study, results indicated that older people are vulnerable to more than one chronic condition and co-morbidity of chronic diseases can have an even more serious impact on HRQoL. China is an aging country [40]–[42]. Therefore, it is essential for China to explore the aging process and the effects of combinations of chronic conditions. This may be one of the most pressing challenges in the prevention and control of chronic diseases. We suggest that the focus of a national chronic disease prevention and control strategy should encompass the young population as well in order to reduce the eventual impact on health.

The MOH has stated that the township hospitals and community health care centers are responsible for the management of hypertension, type 2 diabetes mellitus, and severe mental diseases. It has also established many national chronic disease prevention and control experimental areas across the country [14], [43], [44]. It will be necessary to include the EQ-5D in the indicator framework for surveillance and evaluation of these areas to increase the cost-effectiveness of intervention programs. We did not choose weights from Japan or Hong Kong to calculate the EQ-5D index though these populations are from Asia, because the reliability and validity has not been demonstrated in a large-scale Mainland Chinese population. Standards for the calculation of EQ-5D index for Chinese populations must be developed before it can be used in a wide or in-depth scale.

This is the largest population-based survey of chronic diseases on HRQoL ever conducted in this region. The random multi-stage stratified cluster sampling method was used to ensure sample representativeness. HRQoL was measured using instruments that are widely used throughout the world. Results established an HRQoL baseline for the overall population and the chronically diseased population in Shaanxi Province. We have found that the main determinants of HRQoL include chronic conditions, co-morbidity, age, employment status, education level, marital status, area and location of residence, medical insurance, and level of physical activity. This indicates that improvements in HRQoL will involve consideration from medical professionals, policy makers, society, and the individual. Through the comparison of the effects experienced by those with different chronic conditions, we identified cerebrovascular disease, cancer, and mental disease as the three leading chronic conditions associated with low HRQoL scores. Some limitations must be addressed here. The data were derived from a cross-sectional survey, so the temporal sequence of chronic conditions and changes in HRQoL must be assessed in further studies using a longitudinal design. Another limitation was that EQ-5D scores were replaced with EQ VAS scores, which may be affected by participants' mental status at the time of measurement due to the absence of calculation standards.
